# Protocol for a randomised controlled trial of a family strengthening program to prevent unhealthy weight gain among 5 to 11-year-old children from at-risk families: the Strong Families Trial

**DOI:** 10.1186/s12889-022-13452-x

**Published:** 2022-06-18

**Authors:** Cristy Brooks, Catherine Helson, Madalyn McCormack, Louise A Baur, Timothy Gill, Julie Green, Baki Billah, Paula Cronin, Anoop Johar, Jennifer Plaskett, Michelle Nolan, Monika Latanik, Andre M N Renzaho

**Affiliations:** 1 Translational Health Research Institute, School of Medicine, Western Sydney University, Sydney, NSW Australia; 2 School of Public Health and Preventive Medicine, Monash University, Melbourne, VIC Australia; 3 Sydney Medical School, University of Sydney, Sydney, NSW Australia; 4 Murdoch Children’s Research Institute, Parkville, VIC Australia; 5 Department of Paediatrics, University of Melbourne, Parkville, VIC Australia; 6 Department of Social Sciences, Western Sydney University, Sydney, NSW Australia; 7 University of Technology, Sydney, NSW Australia; 8 Western Sydney Local Health District, Sydney, NSW Australia

**Keywords:** Obesity, Lifestyle, Disadvantage, Family, Children, Weight gain, Culturally diverse, Parenting

## Abstract

**Background:**

Obesity is an increasing health concern in Australia among adult and child populations alike and is often associated with other serious comorbidities. While the rise in the prevalence of childhood obesity has plateaued in high-income countries, it continues to increase among children from disadvantaged and culturally diverse backgrounds. The family environment of disadvantaged populations may increase the risk of childhood obesity through unhealthy eating and lifestyle practices. The Strong Families Trial aims to assess the effectiveness of a mixed behavioural and lifestyle intervention for parents and carers of at-risk populations, i.e. families from culturally diverse and disadvantaged backgrounds, in preventing unhealthy weight gain among children aged 5 to 11 years.

**Methods:**

Eight hundred families from low socio-economic areas in Greater Western Sydney, NSW, and Melbourne, VIC, will be recruited and randomised into a lifestyle intervention or control group. The intervention comprises 90-minute weekly sessions for 6 weeks (plus two-booster sessions) of an integrated, evidence-based, parenting and lifestyle program that accounts for the influences of family functioning. Primary (anthropometric data) and secondary (family functioning, feeding related parenting, physical activity, consumption of healthy foods, health literacy, family and household costs) outcome measures will be assessed at baseline, immediately following the intervention, and 12 months post-intervention.

**Discussion:**

This study will elucidate methods for engaging socially disadvantaged and culturally diverse groups in parenting programs concerned with child weight status.

**Trial Registration:**

This study is registered with the Australian New Zealand Clinical Trials Registry (ACTRN12619001019190). Registered 16 July 2019.

## Background

Childhood obesity is a serious public health problem that tracks from early childhood into adulthood [1, 2]. While the prevalence of childhood obesity has plateaued in most high-income countries, [3] it continues to increase among children from disadvantaged and culturally diverse backgrounds [4–6]. Little attention has been given to trialling interventions to reduce unhealthy weight gain among children from disadvantaged and culturally diverse backgrounds, [7, 8] despite data suggesting that even small reductions in body mass index (BMI) are associated with improvements to cardiovascular risk factors in children [9]. This is a significant issue, since children living in areas with the lowest socio-economic status (SES) are at higher risk of being affected by overweight or obesity than those living in higher SES areas [10–12]. A systematic review of 45 studies found that social disadvantage was associated with increased odds of childhood obesity. Specifically, there was an identified inverse association between adiposity in children and SES in 19 studies (42%) [13]. Moreover, the magnitude of associations between SES and adiposity were further analysed in 24 studies, in which 21 of those found a significant inverse SES-adiposity association [13]. The odds ratio for adiposity in lowest SES children, when compared to highest SES children, ranged from 1.3-6.7, with a median odds ratio of 2.04 [13].

It has been postulated that the family environment of disadvantaged populations is one of several behavioural factors that increase the risk of obesity through unhealthy eating and lifestyle practices [14–17]. The impact on weight status of physical inactivity and corresponding high levels of sedentary behaviour, high energy density diet and other poor dietary and lifestyle choices are well documented, [18–20] and these factors are a particularly impacted by the family environment [14, 15]. Disadvantaged households are more likely to experience conflict, communication difficulties and disengagement, which collectively may affect children’s health including body weight [14]. Therefore, health behaviours are learned within, and shaped by, the family environment. For example, previous studies have shown that family functioning is independently associated with obesity among parents and their offspring, with the prevalence of poor family functioning increasing from 22% to 29% and as high as 39% as the number of obesity risk behaviours increases from 0 to 1 and then 2 or more, respectively [14, 15]. Family social disadvantage affects children’s weight by restricting access to financial, social and educational resources that support children’s healthy development and impacts family functioning [14, 15]. Moreover, low family cohesion, high family conflict, and inadequate social support make it difficult for parents to provide optimal environments for shaping healthy eating habits and physical activity [21]. Consequently, parents have difficulty managing family meals and routines, healthy lifestyles, parenting roles, communication with family members and maintaining social supports [14, 15]. Previous work in this area identified that poor family functioning and parental psychological distress were significantly associated with increased consumption of unhealthy foods among primary school children [21]. It has been estimated that poor family functioning accounts for 14–24% of the variance in BMI among children and adolescents in disadvantaged populations [14, 15].

Behavioural parenting interventions are grounded in learning theory with a focus on teaching parents how to respond empathically to children’s needs [22, 23]. Such approaches result in improved parenting, an understanding of risk and protective factors for children’s healthy development, and increased family cohesion [14]. Behavioural parenting interventions can be alternative approaches to obesity prevention when they address the family environment’s influence upon child weight status, and have potential for translation into health and community service delivery [14]. Findings from such interventions targeting infants and pre-school children, and aiming to improve eating and physical activity patterns, suggest that family focused interventions may also have potential for prevention of unhealthy weight gain, [24–26] with significant intervention effects for a number of maternal feeding practices and child BMI-for-age Z-score (BMIz score) [27]. Studies on fundamental family dynamics of communication, conflict resolution and parent–child engagement are scarce. To date, there have been limited Australian trials of family-focused obesity prevention programs among disadvantaged multi-ethnic families for primary school- aged children.

A series of pilot studies were conducted, which focused on African migrant families with children aged 12-17 years (Healthy Migrant Families Initiative), [17, 28] featuring a two-part intervention. The first focused on healthy eating, active living and healthy body weight, and the second focused on parenting, communication and problem solving [17]. The pilot studies were favourably received as an obesity prevention program among African migrant communities and provided the foundation for the present study. The pilot data was also consistent with other data from Australia and the United States, indicating that children over 5 years of age have a higher prevalence of overweight and obesity compared to children under 5 years [12, 29]. Specifically, the obesity prevalence in the United States among 6- to 11-year-olds in 2017-2018 was 20.3% compared to 13.4% among 2- to 5-year-olds [29]. In Australia, more than 1 in 5 children (24%) aged between 5 and 16 years of age were overweight or obese in 2018 [12]. Furthermore, overweight and obesity prevalence is slightly higher among 5- to 11-year-olds (25.1%) compared to 12- to 16-year-olds (22.5%) [12]. The age group of children prior to adolescence also represents a time of relative stability in a child’s life, when parents are still the major providers of a child’s eating and physical activity environment. For example, in children under 12 years of age approximately 70% of a child’s food is consumed in the home environment and therefore constitutes a major determining factor of a child’s weight trajectory over time [30]. Family functioning, parenting skills training, positive family relationships, healthy lifestyle (nutrition and physical activity) were the key intervention priorities identified. Therefore, the current trial builds on previous research and focuses on evaluating the effectiveness of an integrated package of parenting and lifestyle interventions in preventing unhealthy weight gain and improving the family environment among disadvantaged populations that carry the highest burden of childhood overweight and obesity. It also aligns with advice from the World Health Organization and United Nations Children’s Fund, that acknowledges that reduction and prevention of overweight and obesity in children and adolescents requires a comprehensive and supportive approach, targeting lifestyle and behavioural change [31, 32]. The trial is timely because the COVID-19 and pandemic lockdowns have had an impact on family functioning, intergenerational communication, social isolation and feelings of loneliness [33]. Moreover, such impacts of COVID-19 and lockdowns have been associated with decreased physical activity, poorer nutrition, rises in screen behaviours and food insecurity and shortages, which are estimated to be reversing the obesity plateauing gains [34].

The primary hypothesis is that improving family functioning among parents or carers of 5 to 11-year-old children from disadvantaged backgrounds will reduce BMIz score immediately following the intervention, and 12-months post-intervention, relative to a control group. The secondary hypotheses (immediately following the intervention and 12-months post-intervention when compared to the control group) include:

- The percentage of intervention group families functioning poorly and experiencing parenting-related intergenerational conflicts will be decreased
- The percentage of intervention group families eating a family meal together and adopting healthy dietary practices will be increased
- Among parents and their 5 to 11-year-old children, the mean duration of time spent in physical activity will increase, and screen viewing time will decrease
- The percentage of intervention group parents with adequate knowledge regarding healthy lifestyles (healthy food and dietary choices, healthy eating, physical activity, and sedentary behaviours) will increase
- The self-efficacy of these behaviours in children aged 5 to 11 years will be greater in intervention families than the control group following the intervention program

## Methods

### Study design

**Fig. 1:**
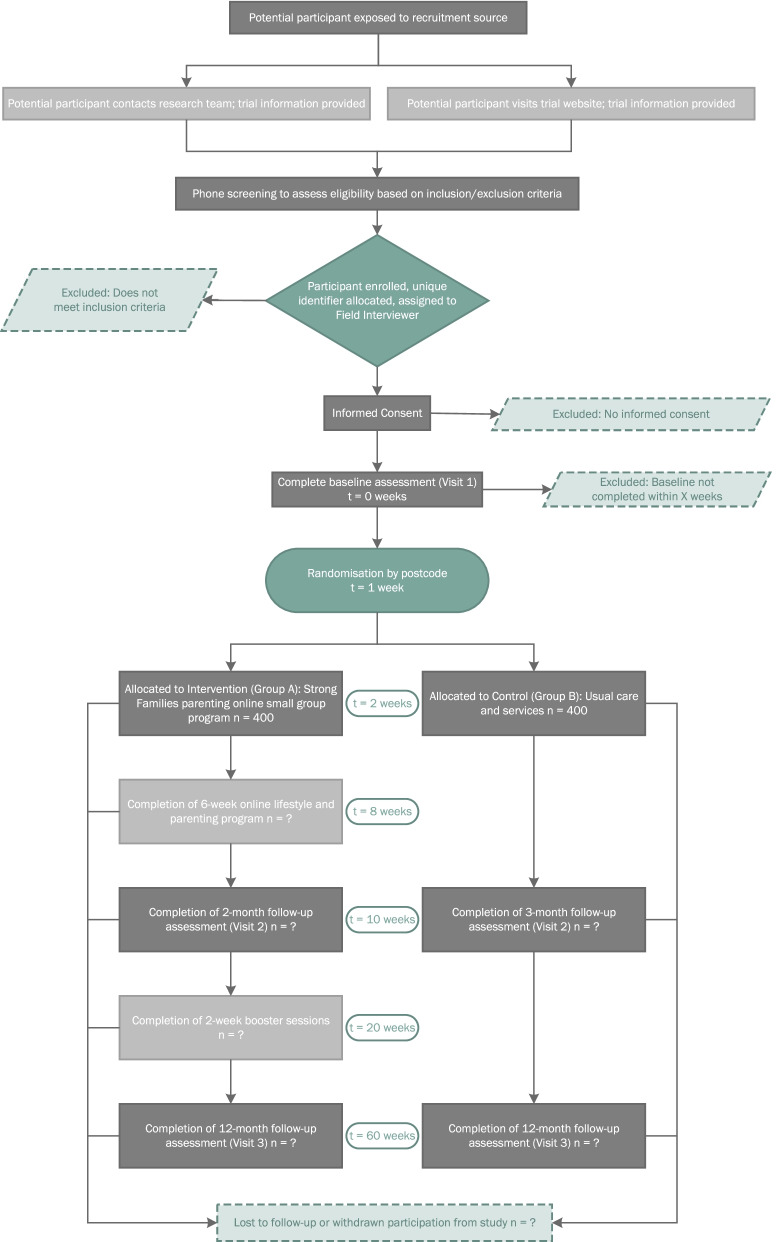
Study design and schedule of enrolment, interventions, and assessments for the Strong Families Trial

The Strong Families Trial will employ a cluster randomised control trial (RCT) design to evaluate the effectiveness of an integrated package of parenting and lifestyle interventions parents and carers of 5 to 11-year-old children in preventing unhealthy weight gain and improving the family environment among disadvantaged culturally diverse populations. The study will evaluate whether a behavioural parenting and lifestyle program consisting of 6 x 1.5-hour weekly group sessions (plus 2 boosters at 3 months post intervention) is effective, sustainable and cost effective in improving children’s anthropometric outcomes. Measurements will be made at baseline (0 weeks), at the end of the 6-week intervention (10 weeks), and at 12-months post-intervention (60 weeks). Details of the study timeline and an overview of methods, randomisation and allocation to groups are shown in Fig. 1 and outlined in the following sections. Briefly, potential participants will be directed to a survey online (trial website), where they can register their interest in the study. Eligible participants will then undergo phone screening to further assess their eligibility and willingness to partake in the Trial. Once enrolled, participants will be assigned to a bilingual field worker for baseline testing and then randomly assigned to the intervention or control group.

### Design and study setting

The trial will use a cluster RCT design with the primary unit of randomisation being postcode of address. This community-based cluster RCT trial will be conducted in the most disadvantaged areas of Greater Western Sydney and Greater Melbourne, with Index of Relative Socio-Economic Disadvantage (IRSD) score of <1000, the cut-off used to indicate socio-economic disadvantage [35]. IRSD is one of four indices of the Socio-Economic Indexes for Areas (SEIFA), developed by the Australian Bureau of Statistics as a measure of socio-economic conditions according to geographical areas. The indices are derived from information obtained during the five-yearly national Census [36]. As at the 2016 Australian population census, the project area has 398 postal areas (POAs) in Greater Western Sydney [37] and Greater Melbourne [38] with a usual resident population of 6,812,614 [39]. Of those, 105 POAs have an ISRD <1000 with a usual resident population of 2,502,846. Of the 105 POAs, 92 have at least 1% of the population speaking Chinese, Arabic, Hindi, Vietnamese, Hindi, or Punjabi at home [40]. In the 92 POAs, 29.6% of households (*n*=131,180) in Greater Western Sydney and 25.2% of households (*n*=120,411) in Greater Melbourne are families with children under 15 years of age [41]. These 92 postcodes will be purposively sampled and included in the study as the targeted population, on the basis of ISRD <1000, >1% of the population speaking one of the languages of interest at home, and having a significant percentage of families with children under the age of 15.

### Participants

Study participants will include 800 families (children aged 5-11 years and their parents/carers) at risk of unhealthy weight gain.

### Recruitment

Methods of recruitment employed in the trial will include flyer distribution, school recruitment, community databases and social media campaigns. The trial flyer will be delivered in each of the languages included in the study (English, Arabic, Vietnamese, Chinese, Hindi and Punjabi) and will be distributed by local community contacts working closely with communities of interest either electronically via email or face-to-face through hard copies. Primary schools (government and non-government) in the study area will be engaged to promote the trial to their school population. Schools will be provided with the trial flyer and a condensed excerpt suitable for inclusion in a newsletter. Approval will be sought from the relevant education bodies. Social media platforms (Facebook, Instagram) will also be used for recruitment purposes, through a paid social media campaign within Western Sydney University. The advertising will target parents from relevant geographical areas with children between 5- to 11-years-of-age and culturally diverse parents of interest. The social media campaign will direct interested participants to the study website (www.westernsydney.edu.au/strongfamiliestrial) for further information. The website will serve as the primary method for interested participants to receive information about the study and submit an expression of interest in participating. Other forms of recruitment that will be used include promoting the study via email and trial flyers in community healthcare centres, contacts and noticeboards; health professionals such as family medical practitioners, paediatricians and dietitians; language or migrant resource centres; and places of worship.

The study will use the Research Electronic Data Capture (REDCap) [42, 43] licensed software to assess the eligibility of interested participants, confirm participant details, track progress and manage participant data. Potential participants enquiring about the study will fill in an online survey for screening purposes through the REDCap software, which will then be followed up by phone screening with research staff to assess eligibility for the study and record participant contact and personal details on the eligible adult and child. Once accepted into the study, the participant will be assigned to a bilingual field worker for baseline assessment.

### Eligibility

Potential participants will be screened for eligibility according to the following inclusion and exclusion criteria:

#### Inclusion criteria:

- Families (parent- or carer-child dyad) who live in the Greater Western Suburbs of Sydney or Western and Northern Suburbs of Melbourne
- Parents who live in the same household as their child aged between 5 to 11 years
- Families who are socio-economically disadvantaged (<1000 Index of Socio-Economic Disadvantage)
- For households with two or more eligible children, the child who had the most recent birthday will be included.

#### Exclusion criteria:

- Self-reported mental or major physical illness or intellectual disability among parents/carers and/or their eligible offspring which would hamper effective participation and/or lead to inability to commit to the group process.

### Outline of trial procedure

Families (parent- or carer-child dyad) with 5 to 11-year-old children in selected suburbs of Greater Western Sydney and Greater Melbourne will be identified, screened and randomly allocated to behavioural parenting and lifestyle program intervention or control. Measurements at baseline (timepoint 1; T1 = 0 weeks), end of intervention (timepoint 2; T2 = 10 weeks) and 12 months post-intervention (timepoint 3; T3 = 60 weeks) will assess changes in children’s weight, height, physical activity, and eating behaviours, as well as in family functioning, parental knowledge and behaviour. Participation in the study will be entirely voluntary and participants will be free to withdraw at any time.

### Assessment

Children’s anthropometric data (primary outcomes) will be measured at 0, 10 and 60 weeks by bilingual field workers to assess the changes weight gain. Measurements will include the child’s height and weight according to a standardised protocol. All bilingual field workers will receive appropriate training by research staff prior to collecting data from research participants. Each field worker will also receive an equipment kit with a detailed instruction manual, height and weight measurement guide, and other necessary items to collect participant data. Parents or carers (of the parent- or carer-child dyad) will participate in a face-to-face interview with trained bilingual field workers to collect data on family functioning, feeding related parenting, physical activity and consumption of healthy foods of the participating child, parents’ health literacy, and family and household costs (secondary outcomes). The following outcome measures will be collected via face-to-face survey using Qualtrics software (Qualtrics, Provo, UT):

1. Study specific questionnaire to capture socio-demographic data
2. Family Functioning using the McMasters Family Assessment Device [44].
3. General Parenting Questionnaire as a composite measure of general parenting behaviours/practices and constructs (sense of competence/efficacy)
4. Feeding related parenting will be measured using the Child Feeding Questionnaire [45].
5. Child's Health-related quality of life will be measured by the Child Health Utility 9D [46–49].
6. Parent-reported participation in sports and other physical activity, measured using 1 item from the NSW Population Survey 2019 [50].
7. Child adherence to physical activity guidelines will be assessed using a composite measure of parent-reported accumulated moderate to vigorous physical activity (MVAP) of at least 60 minutes per day in 2 reference periods (past 7 days and a typical week).
8. Leisure-time sedentary behaviours of children will be assessed using parent-reported time spent in sedentary recreational screen time (i.e. television, seated electronic games and electronic device use other than for schoolwork) in a typical week. It will be a composite measure using 1 item from the Sax Institute Short survey instruments for children’s diet and physical activity, [51] and 1 item from the Youth Risk Behaviour Surveillance System [52].
9. Consumption of healthy foods will be measured by items from the Child Component NSW Population Health Survey 2007-2008, [53] National Health Survey 2017-2018 [54] and Short survey instruments for children’s diet (the Sax Institute for the NSW Ministry of Health) [51].
10. The Strengths and Difficulties Questionnaire [55] will be used to measure socio-emotional problems.
11. Parental health knowledge will be measured using the Lifestyle & General Nutrition Knowledge Questionnaire [56].
12. Participant Evaluation Questionnaire as a composite measure to assess satisfaction with intervention.

### Equipment

Certified NAATI-approved translators (Ethnolink, OPAL Translation Pty Ltd) will be used for the translation of the intervention written materials into the five (5) culturally appropriate languages predominantly spoken in the target geographical areas of interest (Chinese, Arabic, Hindi, Vietnamese, Hindi, and Punjabi), as described earlier.

Digital weight scales (Seca Clara 803) will be used for body weight measurement of participating children. Portable stadiometers (Charder HM200P Portstad) will be used for the measurement of height of participating children. Outcomes measures will be collected and recorded on a portable tablet computer (Lenovo Tab E7, Lenovo PC HK Limited, Bratislava, Slovakia).

### Sample size determination

A minimum sample size was calculated on the statistical power required to detect a difference in standardized BMIz score of 0.15 (SD:0.8) with 80% power and 5% significance level. Taking into account a 19% dropout at end of the intervention and an 11% dropout at follow-up as established in our pilot study, [17] the required sample size is 800 participants after accounting for clustering (i.e. 800 parent- or carer-child dyads) (400 in intervention and 400 in the control group).

### Randomisation / group allocation

The trial will use a cluster RCT design with the primary unit of randomisation being postcode. Each of the 92 postcodes within the study areas will be allocated to an intervention or control arm according to a randomisation schedule generated by the trial biostatistician. The allocation ratio between the intervention arm and the control arm is 1:1.

### Blinding

All bilingual field workers will be blinded to randomisation and will therefore have no knowledge of which group (intervention or control) the participating parent- or carer-child dyad has been allocated to. Group facilitators are unable to be blinded to randomisation given the nature of the intervention but will only be assigned after baseline data collection has been completed. Furthermore, they will take no part in data collection and will only be involved in the delivery of their designated intervention modules. Research staff will not take part in data collection.

### Intervention

Consenting participants nested in the postcodes randomised to the intervention arm will receive the intervention as soon as possible after registration. The Strong Families program (the intervention) will be delivered in two stages: A 6-week group program consisting of 6 x 90-minute online sessions covering both healthy lifestyle and parenting modules. The online sessions will be facilitated by bilingual group facilitators with backgrounds in nutrition, dietetics, psychology or social sciences. The modules are designed to provide participants with new knowledge about healthy eating and physical activity (healthy lifestyle modules) and then teach them skills required to effectively implement this knowledge (parenting modules). Then, approximately 3 months after the weekly sessions, participants will attend 2 x 45-minute online booster sessions. The booster sessions are designed to reinforce and enhance the knowledge and skill development acquired in the weekly sessions. These sessions are also intended to allow participants to evaluate the family environment and troubleshoot any challenging areas. Sessions focus on establishing a welcoming and safe environment, and active learning approaches that maximise engagement in knowledge building of healthy habits, making healthy choices, building stronger families and positive family functioning as well as helpful communication strategies that are sensitive to culturally diverse families living in Australia.

The first three (1-3) modules will focus on creating healthy lifestyle choices as a family but particularly in reference to the participating child. This includes concepts such as reducing screen time and sedentary behaviours, making healthy food choices and increasing physical activity. The second three (4-6) modules focus on strengthening the family dynamic through parenting advice and guidance. Specifically, topics discussed include physical, emotional and social development of children, clear and effective boundaries and communication, understanding behaviour and consequences, and managing emotions. The two booster sessions will be revisions of materials previously discussed in the modules and the ensure understanding. Booster A will revise healthy lifestyles and review understanding of healthy food and lifestyle choices. Booster B will review parenting messages covered and provide guidance on maintaining progress in parental decision-making for a stronger family dynamic.

A detailed outline of each module and booster session is presented in 1. Group facilitators will be qualified bilingual health professionals such as dieticians, psychologists, social workers, counsellors; or bilingual and bicultural workers with previous experience in domains such as family support, community health education and health promotion. They will receive all materials to deliver the intervention from the research team, as well as a detailed instruction manual. All group facilitating staff were required to undergo appropriate recruitment and interview processes prior to employment.

**Table 1:** Program Content of the ‘Strong Families Trial’ intervention

Type of Session	Session Number	Module Title	Objectives	Key Messages	Activities
Healthy Lifestyle Modules	Module 1	Building Healthy Habits	• Welcome participants, provide an opportunity for introductions and explore participant expectations and motivations for the program. • To promote a safe and supportive environment with which participants can actively engage. • To orient participants to the program. • To define key concepts. • Introduce the key healthy lifestyle messages. • To promote healthy drinking habits in children. • To promote physical activity as a part of a healthy lifestyle. • To encourage a reduction in sedentary behaviours. • Promote the importance of sufficient sleep each day for children.	• Strong families can be healthy together by following healthy lifestyle habits every day. • Choose water as a drink. • Be active every day for at least 60 mins. • Decrease screen time to move more and sit less. • Sleep plays an important role in your child’s health.	1.1 Welcome & introductions • Welcome • Session overview • Activity 1: Getting to know each other • Our group rules 1.2 Strong families • Program structure • Strong, healthy families • Healthy lifestyle habits 1.3 Choose water as a drink • Drink water instead of soft drink, juice or cordial • Activity 2: How much sugar • Tips for kids to drink more water 1.4 Getting active each day • Activity 3: Shake it out • Getting active every day • Active families 1.5 Switch off screens • Healthy screen use • Managing screen time 1.6 Sleep • Get enough sleep • Healthy sleep habits 1.7 Wrap-up • Key messages • Take-home activities • Questions • Feedback
	Module 2	Healthy Eating Every Day	• Provide an overview of the session and recap key messages from previous session. • Introduce participants to concept of ‘everyday’ foods and ‘sometimes’ foods using the Australian Guide to Healthy Eating (AGHE) and the five food groups. • Highlight the main nutrients & portion sizes for each of the five ‘everyday’ food groups and promote the benefits of healthy eating. • Increase participants understanding that ‘sometimes’ foods should be limited as they are high in fat, salt & sugar. • Introduce positive parenting feeding practices to promote development of healthy eating habits. • Encourage participants to enjoy breakfast each day. • Promote the importance of a healthy lunch box and snack choices and encourage increased intake of fruit & vegetables through these choices.	• Eat ‘everyday’ foods from the five food groups. • Increase ‘everyday’ foods and limit intake of ‘sometimes’ foods. • Children need a balance of foods and drinks for optimal growth and development. • Strong, healthy families enjoy eating together. • Breakfast is a great way to start the day. • Healthy lunchboxes and snacks contain ‘everyday foods’.	2.1 Introduction • Welcome • Module 2 overview • Recap - Module 1 2.2 Understanding healthy eating • ‘Everyday’ & ‘sometimes’ foods • Healthy fats • ‘Sometimes’ foods 2.3 Healthy eating habits • Building healthy eating habits 2.4 Good start to the day • Why is breakfast important? • What is in a healthy breakfast? • Breakfast helpers 2.5 Healthy lunch & snack habits • Healthy lunch & snack habits • Lunchbox & snack helpers • Activity 1: ‘Everyday’ lunches & snacks 2.6 Wrap-up • Key messages • Take-home activities • Questions • Feedback
	Module 3	Making Healthy Choices	• Allow participants to reflect on prior learning and to provide an overview of the module • To encourage limited intake of takeaway foods with high sugar, salt and fat through healthier food choices. • For parents to develop skills to enable them to modify recipes into healthier options. • For parents to be able to confidently read food labels & nutrition symbols to inform healthy choices. • To highlight that pre-planning can help make healthy food purchases and that healthy eating need not be expensive	• Simple swaps make favourite meals healthier • Understanding food labels can help you make healthy food choices at the supermarket. • Healthy food choices can be easy, convenient and affordable.	3.1 Introduction • Welcome • Module 3 overview • Recap - Module 2 3.2 Smart food swaps • Smart swaps for cooking • Healthier cooking methods • Healthy choices away from home • Activity 1: Recipe makeovers 3.3 Understanding food labels • How to read a food label • Nutrition information • Ingredients • Front of pack labelling • Activity 2: Food detectives 3.4 Smart supermarket choices • Isn’t healthy eating more expensive? • Healthy shopping habits • Supermarket savvy • Combating pester power 3.5 Wrap-up • Key messages • Take-home activities • Questions • Feedback
Parenting Modules	Module 4	Building Strong Families	• Provide an overview of the module and highlight the importance of family functioning on a family’s health • Explore participant’s expectations and experiences of becoming parents • Introduce the essential components of a strong family • Develop an understanding of the sequence of physical, emotional, social and cognitive changes that occur in children	• Our expectations and experiences of becoming a parent have been shaped by many things including our upbringing, values, family and cultural influences. • During childhood your child develops physically, emotionally, socially and cognitively. The relationship with their family plays an important role in supporting this development. • Children need strong families in order to thrive. • A strong family has emotional and physical security, lots of warmth, care and positive attention, firm, fair rules and boundaries, good communication and connections to others outside of the family.	4.1 Introduction • Welcome participants • Introduction to Module 4 4.2 Becoming a parent • Thoughts about becoming a parent 4.3 Essential components of a strong family • Strong Families • Activity 1: Bullseye 4.4 Understanding childhood development • Child developmental milestones 4.5 Wrap-up • Key messages • Take-home activities • Questions • Feedback
	Module 5	Praise, Rewards, Rules & Consequences	• Highlight the role of positive attention, praise and rewards in promoting good behaviour • Promote the importance of limit setting as a means to guide children’s behaviour • Develop an understanding of how to use consequences as a strategy to manage their children’s behaviour effectively.	• Family rules help create structure and can help guide children to understand what behaviours are okay and not okay. • The way that you respond to your child’s behaviour directly influences whether they are more or less likely to engage in the behaviour again. • Physical punishment (e.g. smacking) is not an effective way to help children to follow rules.	5.1 Introduction • Welcome • Introduction to Module 5 5.2 Promoting positive behaviour • The importance of praise • Tips for using praise • Rewards 5.3 Setting family rules • Family rules – what are they and why are they important? • Developing family rules • Following the rules: what to expect 5.4 Responding to children’s behaviour • Smacking- It is never okay • Using consequences effectively • Activity 1: How would I respond? 5.5 Wrap-up • Key messages • Take-home activities • Questions • Feedback
	Module 6	Improving Communication & Managing Emotions	• Explore challenges in communication and provide parents with tools for more effective communication. • Gain an understanding of how to support children to identify and manage their emotions effectively. • Equip parents with strategies to manage stress, highlighting the importance of self-care and support networks. • Explain the importance of chores.	• The way that we communicate with others has a significant impact on the quality of our relationship and on the outcomes of our interaction. • Emotions such as joy, sadness, fear, anger and disgust can occur daily. It is important to find healthy ways to express our emotions. • Stress is a normal part of life and is experienced by everyone from time to time. However, when stress is frequent or severe, it can impact on your quality of life and therefore it needs to be effectively managed. • Self-care can enhance your health and wellbeing and is vital in helping you care for others. • Assigning chores to family members can help reduce stress, while supporting children to develop essential life skills.	6.1 Introduction • Welcome • Introduction to Module 6 6.2 Improving family communication • Activity 1: Follow the instructions • Communication skills for your family • Teaching children how to communicate 6.3 Understanding emotions • The importance of understanding emotions • Activity 2: Identify the emotion • Helping children to deal with big emotions 6.4 Reducing stress • The basics of stress 6.5 Sharing the load • Chores for children 6.6 Wrap-up • Program recap • Activity 3: Creating a maintenance plan • Wrapping up and Next Steps • Feedback
Booster Sessions	Booster Session A	Healthy Lifestyles	• Welcome participants and introduce them to the first booster session. • Revise and assess participants understanding of the key messages of the healthy lifestyle modules. • Review implementation of Strong Families maintenance plan. • Ensure that participants have a thorough understanding of all key messages by addressing any questions and areas of concern.	• It is important to maintain the progress you have made in the Strong Families program to ensure that your family stays strong and healthy.	A.1 Introduction • Welcome • Introduction to Booster A A.2 Review of Healthy Lifestyle modules •Review of key messages A.3 Positive changes & ongoing challenges •Reflecting on learning A.4 Troubleshooting & wrap up •Troubleshooting •Feedback •Wrap up
	Booster Session B	Parenting	• Welcome participants and introduce them to the second booster session. • Revise and assess participants understanding of the key messages of the parenting modules. • Review implementation of Strong Families maintenance plan. • Ensure that participants have a thorough understanding of all key messages by addressing any questions and areas of concern.	• It is important to maintain the progress you have made in the Strong Families program to ensure that your family stays strong and healthy.	B.1 Introduction •Welcome •Introduction to Booster B B.2 Review of Parenting modules •Review of key messages B.3 Positive changes & ongoing challenges •Reflecting on learning B.4 Troubleshooting & wrap up •Troubleshooting •Feedback •Wrap up

### Control group

Consenting participants nested in the postcodes randomised to the control arm will not receive the lifestyles and parenting intervention, but rather will receive usual care (self-directed access to and use of parenting and child health services). The control group will form the basis of a comparator group of participants to better understand unhealthy weight gain in children with similar characteristics to the intervention group. All study participants will be invited to an overall presentation and summary of the research findings at the conclusion of the study.

### Data management

Data generated in this research will comprise three categories:

#### Study administrative data

Data and documents produced in the conduct of the trial. These include: protocol, master copies of Participant Information Statement and consent forms, signed agreements, master randomisation list, screening logs, enrolment logs. Study administrative data will be collected and stored using standard MS Office software.

#### Research data

Data collected and produced during the intervention phase of the study. These data include participant questionnaire results, signed consent forms, height and weight measurements. Research data will be collected using Qualtrics surveys. Data from Qualtrics Surveys will be extracted in Ms Excel, CSV or PDF formats depending on the type of data (e.g. questionnaire results as MS Excel or CSV, eConsent forms as PDF). Once exported from Qualtrics, working research data will be stored in a shared cloud storage via CloudStor (AARNet), as Excel, CSV or PDF formats depending on the type of data (e.g. questionnaire results as MS Excel or Stata, eConsent forms as PDF). Research data will be managed using REDCap electronic data capture tools hosted at Western Sydney University [42, 43]. REDCap (Research Electronic Data Capture) is a secure, web-based software platform designed to support data capture for research studies, providing 1) an intuitive interface for validated data capture; 2) audit trails for tracking data manipulation and export procedures; 3) automated export procedures for seamless data downloads to common statistical packages; and 4) procedures for data integration and interoperability with external sources. Participants will be assigned a Participant ID, which will be used on all data collection instruments and then de-identified for data analysis. Data will have an access rights assigned to it accessed only by the authorised research team members. Data will be retained for 15 years after the completion of the research. Confidentiality will be maintained by using the process of data cleaning. Researchers will remove identifiers to create a clean set of data. This clean dataset will not contain information that identifies respondents, such as a name or address (such identifying information will be stored elsewhere, in separate, protected files). Some identifiers will be easily recognised and dealt with. For example, the names of respondents will be replaced with pseudonyms & addresses will be deleted from the file once they are no longer needed. Only researchers will have access to the data that is collected and stored. Participant’s identities will not be identifiable in publications resulting from this investigation. All analyses will use the de-identified dataset. The identified dataset will not be shared with non-study personnel. However, de-identified data may be shared with other researchers in accordance with the National Statement on Ethical Conduct in Human Research (2007). Teamup (Teamup Solutions AG, 2014-2021) will be used to manage field workers.

#### Third party data

Data will be obtained from the Services Australia (Medicare Benefits Scheme (MBS) records and Pharmaceutical Benefits Scheme (PBS) records) and from State/Territory health data custodians (public and private hospital admissions, emergency departments, ambulance services, outpatient records). Health administrative data will stored in secure password-protected folders. Best practice file-naming conventions will be used include eliminating spaces from file names by using underscores or CamelCase, including dates in standard format YYYYMMDD, and using clear, consistent descriptors. All MBS & PBS data will be destroyed at the end of the retention period and will not be used for any future, unspecified purposes other than this specific research.

### Project management

The Study project will be managed by a team of researchers across Western Sydney University, Sydney, and Monash University, Melbourne. Research staff will use several methods to manage the project. The primary method of project management will be the REDCap data capture software to collect and manage participant information from recruitment and baseline testing through to randomisation, intervention and follow-up testing. Bilingual field workers and group facilitators of the intervention modules will be communicated with via email, phone and Zoom. Research team members will have regular communication and meetings to maintain consistency and adequate flow of recruitment processes and other tasks. Scheduling of testing sessions for field workers will be assigned and managed using Teamup software.

### Statistical analysis

Data on socio-demographics, anthropometry, general parenting, feeding-related parenting, child’s health and quality of life, physical activity and consumption of healthy foods, parents’ health knowledge and health and household costs will be collected. Child’s anthropometric measurements will be taken at baseline, at completion of the intervention and 12 months after completion of the intervention. Height and weight of the participating child will be used to calculate the BMIz score (primary outcome measure) [57, 58]. Cost per BMIz saved and incremental cost per quality-adjusted life-year gained will also be calculated (Incremental cost per quality-adjusted life-year gained at baseline, end of intervention and 12 months following intervention). Parents will respond to self-administered questionnaire on family functioning, feeding related parenting, physical activity and consumption of healthy foods of the participating child, parents’ health literacy, and family and household costs. The analysis will follow an intention-to-treat approach and with supplementary per protocol analysis. Research data will be analysed with Stata 16 [59]. The statistical analysis will use a modelling approach that aims to capture the profile of the intervention group compared to the control group and while accounting for potential unobserved differences between the intervention and control groups that (if not accounted for) could bias results. The models will evaluate the impact of the interventions over time by testing for an interaction between time and intervention group. This will be followed by multivariable linear models after adjusting for baseline characteristics and other variables (standard demographic variables such as age, sex, income, education, and SEIFA). Subgroup analysis will be undertaken for within-intervention group differences between English speaking and Non-English-speaking groups. Model assumptions will be checked and appropriate adjustments to the analysis made where necessary. A *p*-value <0.05 will be considered statistically significant. The cost-effectiveness analyses will be conducted to determine incremental cost per quality adjusted life year gained by comparing the direct costs and outcomes of behavioural parenting and lifestyle program over usual care from a health care perspective at completion of the intervention and at 12 months post-intervention. The within-trial analysis will adopt a micro-costing approach to calculate the costs of resources used to implement the intervention, including training, staff and travel costs; family or household costs (resources related to food shopping and activities); and health care resources. One of the aims of the project is to undertake economic evaluation to evaluate the effectiveness of the proposed intervention for parents or carers from disadvantaged backgrounds, in preventing unhealthy weight gain in children aged 5 to 11 years living in the same household. This requires data linkage to Medicare funded services and dispensing of subsidised pharmaceuticals to capture total health care expenditure and out-of-pocket costs (Services Australia RMS1190). All analyses will be adjusted for clustering and all participant data will be de-identified prior to analysis.

### Adverse events

Before taking part in the study, potential participants will be screened by researchers over the phone based on inclusion and exclusion criteria of the study protocols. In addition, before being admitted into the study, eligible participants will be provided with an overview of the study procedure, the type of information they will asked to disclose and the level of time commitment the study will require. They will also be informed about the randomisation into either the intervention or control group. While we do not anticipate any parts of the intervention will cause distress, potential participants who feel distress regarding any of the topics will be screened out. When they had been deemed eligible to participate, participants will be provided with age-appropriate full information regarding the intervention. Parents/Carers and children will be given ample time to ask any questions before obtaining written informed consent. It will also be emphasised that they will not be identified in any report and that all their answers will be kept confidential - accessed only by authorised researchers and information obtained will only be used for the purpose of this research. Further, they will be reassured that they are able to choose not to answer questions asked in the study should they find these questions uncomfortable. They will also be reminded they can withdraw from the study at any time without affecting their relationship with the researchers, the University or its affiliates. Interviewers will be instructed to be vigilant about interviewees emotional response to questions asked. Should any of the questions generate an emotional response from interviewees, they will be asked if they want to have a break, postpone or terminate the interview and be referred to appropriate counselling services, a list of which is included in the participant information sheet. As the project's participants are children/families from migrant communities and low socio-economic areas, we will adopt culturally respectful approaches and considerations, but will not compromise the child’s safety and wellbeing. The project will follow the principles outlined in the United Nations' Convention on the Rights of the Child (1989) [60], which has been adopted by the Australian Human Rights Commission. Researchers will also assure children that any data collected will be kept confidential, which is important because of the power imbalance between children and adults. However, if any disclosure of mistreatment or inappropriate behaviour are made during the trial by the parent/carer or child, participants will be informed that the researchers are not equipped to deal with such disclosures and will be provided with contact details to the relevant authorities whose purpose is to deal with such matters. As the trial is being conducted over two states NSW and Victoria, if the need arises participants in NSW will be directed to contact Family and Community Services and participants in Victoria will be advised to contact the Department of Health and Human Services. Kids Helpline materials will also be provided to all field workers to distribute among participants as needed. All bilingual field workers will have Police and Working with Children Checks, will not be alone with a child at any point, and will only complete testing of outcome measures when both a child and their parent/carer is present to avoid any safety concerns.

## Discussion

The Australian Commonwealth and state governments are enthusiastic about trialling and adopting new approaches to addressing child obesity, increasing the likelihood that benefits of the intervention can be translated more widely. This project addresses 3 of the 4 goals in the National Research Priority area of Promoting and Maintaining Good Health: preventing unhealthy weight gain in childhood years (‘preventive healthcare’ and ‘a healthy start to life’) and targeting those who are most vulnerable due to their socio-economic circumstances (‘strengthening Australia’s social and economic fabric’). The study will provide evidence to inform the current National Strategic Framework for Chronic Conditions regarding programs that warrant ongoing support to ensure the most disadvantaged are not left behind in efforts to tackle overweight and obesity. The intervention components have potential for integration into mainstream service delivery, and scalability through health and community services, providing a valuable contribution to the evidence base used in the design of multi-pronged population-based programs, nationally and internationally. The proposed RCT is innovative because it will test the effectiveness of an integrated package of parenting and lifestyle interventions rather than studying the effectiveness of a single intervention (e.g. lifestyle components), providing scientific evidence of the additive effectiveness of a mixed parenting program when combined with a standard lifestyle intervention to prevent unhealthy weight gain and improve the family environment among migrant populations. This study will elucidate methods for engaging socially disadvantaged and culturally diverse groups in parenting programs concerned with child weight status.

## Data Availability

Not applicable.

## Abbreviations

BMI: Body Mass Index
BMIz: BMI-for-age Z-score
IRSD: Index of Relative Socio-Economic Disadvantage
MBS: Medicare Benefits Scheme
POAs: Postal Areas
PBS: Pharmaceutical Benefits Scheme
REDCap: Research Electronic Data Capture
RCT: Randomised Control Trial
SEIFA: Socio-Economic Indexes for Areas
SES: Socio-Economic Status
